# Effects of Local Wound Infiltration Post-Mastectomy Using Bupivacaine Alone, Bupivacaine With Ketamine, and Bupivacaine With Dexmedetomidine: A Randomized Double-Blind Study

**DOI:** 10.7759/cureus.65727

**Published:** 2024-07-30

**Authors:** Priyanka Raj, Nitesh Sinha, Ramesh K Kharwar, Dipali Singh, Sourabh Kumar, Shio Priye, Jay Prakash

**Affiliations:** 1 Department of Anesthesiology, Rajendra Institute of Medical Sciences, Ranchi, Ranchi, IND; 2 Department of Critical Care Medicine, Rajendra Institute of Medical Sciences, Ranchi, Ranchi, IND

**Keywords:** pain management, dexmedetomidine, ketamine, bupivacaine, post mastectomy

## Abstract

Background and aim

While the infiltration of surgical incisions with local anesthetics is not a new practice, it remains a crucial component of contemporary multimodal analgesia protocols. This study aimed to evaluate the efficacy and safety of using adjuvants in combination with local anesthetic wound infiltration for pain management in patients undergoing mastectomy surgery.

Methods

Eighty-one patients aged 18-60 years, classified as American Society of Anesthesiologists (ASA) grade I or II, were scheduled for unilateral mastectomy and randomly assigned to three groups of 27 each. The groups were designated as Group C (bupivacaine alone), Group D (bupivacaine with dexmedetomidine), and Group K (bupivacaine with ketamine). Group C received 0.25% bupivacaine alone, Group D received 0.25% bupivacaine with 1 mcg/kg dexmedetomidine, and Group K received 0.25% bupivacaine with 1 mg/kg ketamine. The time to achieve a Visual Analogue Scale (VAS) score of 3 following local wound infiltration was recorded for each group. Additionally, total postoperative fentanyl intake during the first 24 hours, as measured by the patient-controlled analgesia (PCA) pump, was compared among the groups. Sedation levels were assessed using the Ramsay Sedation Scale (RSS). Data were analyzed using the Chi-Square test and one-way ANOVA in IBM SPSS Statistics for Windows, Version 28.0 (Released 2021; IBM Corp., Armonk, NY, USA).

Results

Demographic factors were similar across the three groups. Analysis of the VAS scores revealed that the ketamine group provided better postoperative pain control than the dexmedetomidine group (p < 0.001). Groups D (71.72 ± 71.73) and K (3.53 ± 13.42) had significantly lower 24-hour fentanyl intake (in mcg) compared to Group C (the control group), as measured by PCA. Additionally, Group C had a significantly lower RSS at the sixth hour (p = 0.003) compared to both Groups D and K.

Conclusion

Ketamine, when used as an adjuvant to bupivacaine for local infiltration, enhances the effectiveness and prolongs postoperative analgesia more effectively than dexmedetomidine in patients undergoing mastectomy.

## Introduction

Managing patients with acute surgical pain poses a difficult task in the perioperative setting. Effective postoperative pain management is associated with higher levels of satisfaction and recovery. Poorly controlled postoperative pain may prolong healing. The multimodal approach is currently the accepted standard for perioperative pain treatment. Several postoperative pain management regimens are available, depending on the type of operation. These regimens use a wide range of drugs, including opioids, non-opioids, paracetamol, alpha-2 agonists, and wound infiltrations, in addition to local anesthetics, muscle blocks, and regional anesthetics [1].

Opioid side effects, including suppression of respiratory drives, pruritus, and associated nausea, necessitate a more informed and convenient approach to postoperative pain treatment in the modern era. Opioid-free analgesia regimes are becoming a cornerstone of pain care in appropriate instances. The efficiency of pain treatment following surgery is based on various aspects, including the frequency of unfavorable events, side effects associated with opioids, and the duration of recovery, including functional recovery [2]. The use of local anesthetics perioperatively can reduce pain to a sufficient degree to provide adequate patient satisfaction. Instilling local anesthetics into surgical wounds during the closure can lessen the need for postoperative analgesics [3].

Ketamine functions as an N-methyl-D-aspartate (NMDA) receptor inhibitor. This inhibition of NMDA receptors is found at both central and peripheral levels. On the other hand, dexmedetomidine functions as a selective alpha-2 adrenergic receptor agonist. It is a centrally acting alpha-agonist with a very high affinity for alpha-2 as compared to alpha-1 adrenergic receptors. Its use has not been limited only to providing sedation but has now gone to the potentiation of local anesthetics. The analgesic effects of dexmedetomidine are amplified when taken intravenously, intramuscularly, intrathecally, epidurally, or perineurally [3].

The purpose of this study was to evaluate the efficacy of adjuvants in combination with local anesthetics when they are deposited on either side of the incision line in mastectomy patients to provide a safe and reliable technique for pain management. Previous studies have investigated the efficiency of local anesthetic infiltration with adjuvants in situations of abdominal hysterectomy; however, it has not been studied in cases of mastectomy. Furthermore, the current study used patient-controlled analgesia (PCA) to determine the overall amount of analgesics consumed.

This study examines the effects of blocking the pain cycle by adding local anesthetics around the surgical incision line for postoperative pain. It was done in patients with unilateral mastectomy using bupivacaine plus dexmedetomidine versus ketamine plus bupivacaine, along with plain bupivacaine as a control.

## Materials and methods

The study was conducted in the Department of Anesthesiology at the Rajendra Institute of Medical Sciences (RIMS), Ranchi, India. It received ethical clearance from the Institutional Ethics Committee of RIMS via letter no. 231, dated 19/05/21. It was prospectively registered under the Clinical Trials Registry - India (CTRI) with registration number CTRI/2021/08/035933. The study included 81 patients aged 18-60 years with an American Society of Anesthesiologists (ASA) grade I or II, scheduled for mastectomy under general anesthesia. Informed consent was obtained from all participants.

Exclusion criteria included known allergies to the study drugs, use of other analgesics within 24 hours of surgery, coagulation disorders, significant cardiorespiratory conditions, kidney or liver diseases, alcoholism, and mental illnesses affecting pain perception and assessment.

Before surgery, patients were briefed about the use of a PCA pump (Fresenius KabiAgilia® SP PCA, Germany) and the Visual Analogue Scale (VAS), which ranges from 0 to 10, with 0 indicating no pain and 10 representing the worst pain imaginable. Each patient was blinded to the study drugs used for pain control. Randomization was achieved by distributing slips of paper with assigned numbers to patients, and double-blinding was maintained by keeping the study substances hidden from both patients and observers.

Groups C, D, and K were formed based on the local infiltration drug administered. Patients in the control group (Group C) received 40 ml of 0.25% bupivacaine. In Group D (the dexmedetomidine group), patients received 40 ml of 0.25% bupivacaine with 1 mcg/kg body weight of dexmedetomidine. Group K patients received 40 ml of 0.25% bupivacaine with 1 mg/kg body weight of ketamine. Twenty milliliters of the study drugs were injected into both sides of the surgical wound.

A standard perioperative regimen was followed for all groups. After verifying patient identity, monitoring equipment, including an ECG, noninvasive blood pressure (NIBP) monitor, and oxygen saturation probe, was attached. Preoxygenation was performed with a mask for three minutes. Anesthesia induction involved IV midazolam (0.25 mg/kg), propofol (2 mg/kg), and fentanyl (2 mcg/kg). Following ventilation confirmation, vecuronium (0.1 mg/kg) was administered for endotracheal intubation. General anesthesia was maintained with N2O, O2, and isoflurane.

Postoperatively, the study drugs were administered locally along both sides of the incision lines. Reversal of neuromuscular blockade was achieved with IV neostigmine (50 mcg/kg) and glycopyrrolate (0.0125 mcg/kg). Post-surgery, respiratory rate, oxygen saturation, heart rate, and NIBP (mean, diastolic, and systolic) were recorded. Pain was assessed using the VAS, and sedation was recorded using the RSS. The time to achieve a VAS score of 3 was recorded for each group. When the VAS score was ≥3, a 0.5 mcg/kg IV bolus of fentanyl was administered. Patients were then connected to a PCA system delivering fentanyl (10 mcg/ml) with a single 1 ml bolus and a 10-minute lockout time. There was no background infusion. The PCA pump was used for 24 hours following the first fentanyl bolus.

Statistical analysis

Statistical analysis was conducted using IBM SPSS Statistics for Windows, Version 28.0 (Released 2021; IBM Corp., Armonk, NY, USA). Continuous variables were expressed as means and SDs. Sample size estimation was performed based on the method outlined by Mohamed et al., which accounts for the assumptions of a one-way ANOVA [3]. The averages and SDs for the three groups were 6.80 mg and 3.19 mg, respectively. The final predicted sample size for each group was 27, determined using a 5% alpha level and 80% power.

## Results

**Figure 1:**
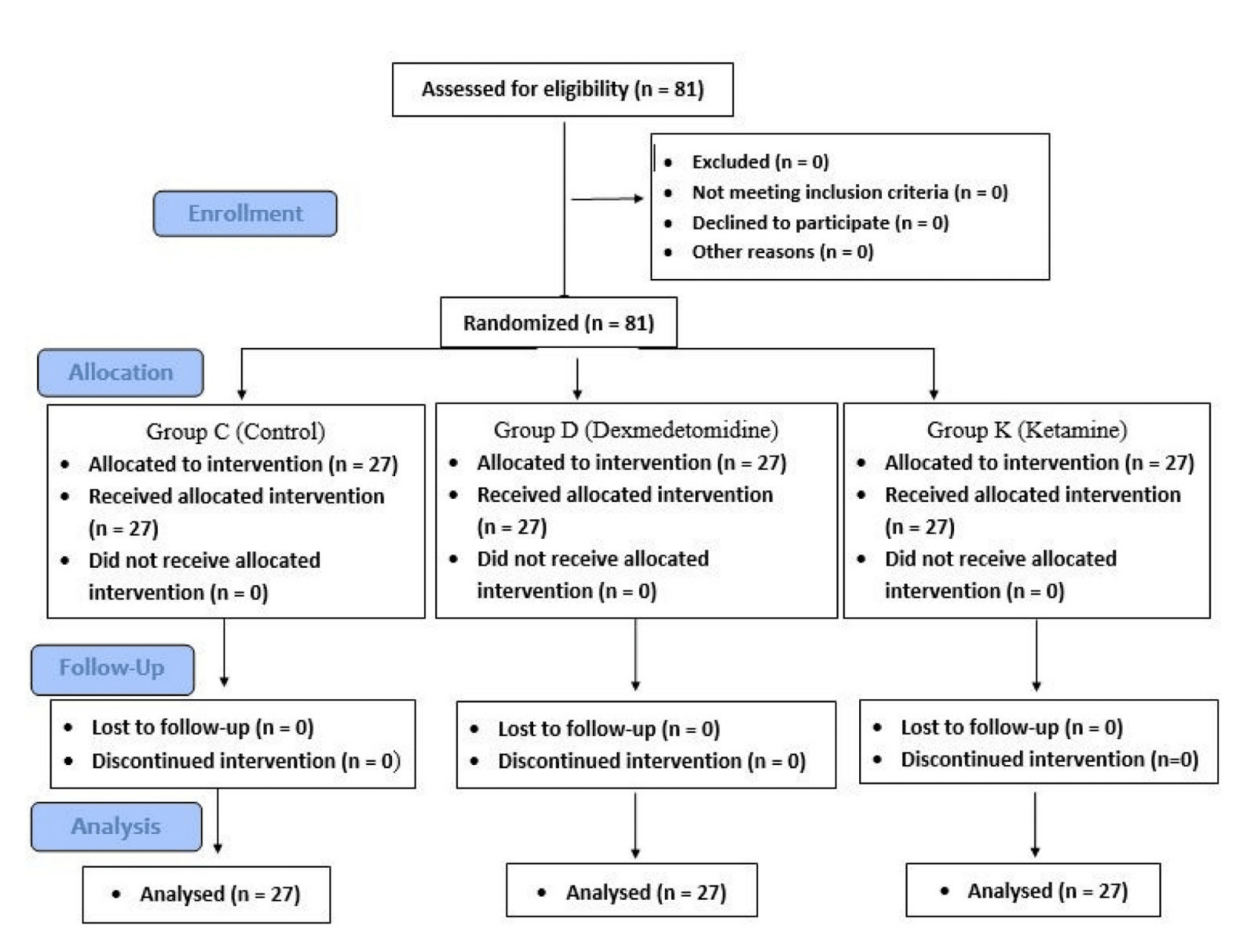
CONSORT flowchart CONSORT, Consolidated Standards of Reporting Trials

The study was completed by a total of 81 patients, as detailed in the Consolidated Standards of Reporting Trials (CONSORT) flowchart (Figure 1).

Regarding patient demographics, ASA physical status, and surgical time, there were no significant differences among the three groups (Table 1).

**Table 1:** Comparison of demographic data Values are presented as mean ± SD. Group C: control; Group D: dexmedetomidine; Group K: ketamine ASA: American Society of Anesthesiologists

Variables	Mean ± SD			p-value
Age (years)	Group C	Group D	Group K	0.837
	46.11 ± 10.412	47.04 ± 10.570	45.37 ± 7.827	
Weight (kg)	56.70 ± 8.189	55.48 ± 6.11	56.56 ± 7.827	0.805
Duration of surgery (hours)	1.93 ± 0.267	1.963 ± 0.308	1.98 ± 0.350	0.809
ASA (I:II)	19:8	21:6	19:8	0.786

The mean time to reach a VAS score ≥3 was significantly shorter in Group C (10.05 ± 6.46 hours) compared to Group D (16.55 ± 6.37 hours) and Group K (19.50 ± 4.94 hours) (Table 2).

**Table 2:** Comparison to reach a VAS score of ≥3 postoperatively (in hours) Values are presented as mean ± SD. Group C: control; Group D: dexmedetomidine; Group K: ketamine VAS: Visual Analogue Scale

	Group C	Group D	Group K	p-value
Mean ± SD	10.05 ± 6.46	16.55 ± 6.37	19.50 ± 4.94	0.002

Groups K and D reported significantly longer durations before the first demand for rescue analgesics compared to Group C. Total fentanyl consumption through PCA over 24 hours was significantly higher in Group C (193.89 ± 79.31 mcg) compared to Group D (71.72 ± 71.73 mcg) and Group K (3.54 ± 13.42 mcg). The total number of PCA pump attempts was also much higher in Group C (60.81 ± 38.34) compared to Group D (22.22 ± 45.32) and Group K (0.37 ± 1.57), with p < 0.05 (Table 3).

**Table 3:** Comparison of the opioid requirements and consumption Values are presented as mean ± SD. Group C: control; Group D: dexmedetomidine; Group K: ketamine PCA: patient-controlled analgesia

Variables	Mean ± SD			p-value
	Group C	Group D	Group K	
First rescue analgesic requirement (hours)	10.05 ± 6.46	16.55 ± 6.37	19.50 ± 4.94	0.003
Total fentanyl consumption (mcg)	193.889 ± 79.31	71.722 ± 71.73	3.537 ± 13.42	0.001
Number of attempts on PCA	60.81 ± 38.34	22.00 ± 45.32	0.37 ± 1.57	0.001

The RSS score was significantly lower in Group C compared to Groups D and K at the sixth hour (p = 0.003) (Table 4).

**Table 4:** RSS score at the sixth hour Values are presented as mean ± SD. Group C: control; Group D: dexmedetomidine; Group K: ketamine RSS: Ramsay Sedation Scale

RSS score	Group C	Group D	Group K	p-value
0	5	9	6	0.034
1	2	4	11	
2	6	7	4	
3	10	6	6	
4	4	1	0	

## Discussion

We compared the efficacy of adding ketamine (Group K) or dexmedetomidine (Group D) to bupivacaine versus using bupivacaine alone (Group C) for local infiltration around the surgical incision in patients undergoing unilateral mastectomy. Our observations revealed that postoperative fentanyl requirements were significantly lower in Groups D and K compared to Group C. This difference was statistically significant. PCA usage indicated that Group C required significantly more fentanyl than Groups D and K over a 24-hour period. Effective and safe postoperative analgesia can be achieved using local anesthetics combined with adjuvants, potentially reducing the upregulation of peripheral nociceptor activity that contributes to increased pain sensitivity [4].

Local anesthetic infiltration at the wound site effectively reduces postoperative wound pain and the need for analgesics in patients undergoing single-incision laparoscopic colectomy [5]. This technique can be a valuable component of a multimodal postoperative pain management plan. While local anesthetic infiltration is effective for pain control, it may not provide long-lasting and profound analgesia. Adjuvants like dexmedetomidine and ketamine, when added to local anesthetics, can enhance the efficacy of field blocks. Dexmedetomidine has been shown to be effective in IV sedation [6], spinal [7], epidural [8], and caudal anesthesia [9], as well as in prolonging the effects of local anesthetic infiltration.

In line with Mohamed et al.’s findings, which reported that adding ketamine to bupivacaine improves analgesia without significant adverse effects, our study supports this conclusion. Their study noted that dexmedetomidine used for local wound infiltration reduced total morphine consumption, delayed initial analgesic requests, and attenuated the stress response without undesirable side effects [3]. While their study used 2 mg/kg of ketamine and 2 mcg/kg of dexmedetomidine with 0.25% bupivacaine, our study used 1 mg/kg of ketamine and 1 mcg/kg of dexmedetomidine. Their study found that the ketamine group required less rescue analgesia (6.80 ± 3.19 mg) compared to the dexmedetomidine group (8.39 ± 3.86 mg) and the control group (13.33 ± 4.01 mg). Our results align with these findings, as Group K (3.54 ± 13.42 mcg) and Group D (71.72 ± 71.73 mcg) required less rescue analgesia (fentanyl) than Group C (193.89 ± 79.31 mcg).

Similarly, Biomy et al. found that ketamine is preferable to dexmedetomidine when added to local anesthetic wound infiltration, as it extended the time to the first analgesic request and reduced overall analgesic use following cesarean section. However, their study used higher doses of ketamine or dexmedetomidine (2 mg/kg) in a 40-ml 0.25% bupivacaine solution [10]. Tverskoy et al. also supported the efficacy of ketamine by showing that adding it to bupivacaine 0.5% improved pain control and increased the potency of local anesthetics in patients undergoing bilateral herniorrhaphy [11]. Although our study used ketamine with 0.25% bupivacaine, the extended duration to reach a VAS score of ≥3 suggests an enhanced analgesic effect.

Kazemeini et al. studied the effects of ketamine versus 0.5% bupivacaine for local infiltration in anal fistula surgery, finding that ketamine reduced postoperative analgesic requirements. Their study used 1 ml of ketamine with 0.5% bupivacaine, whereas we used 1 mg/kg of ketamine with 0.25% bupivacaine. In our study, Group K achieved a VAS score of ≥3 in 19.50 ± 4.94 hours, while their study reported a VAS score of less than 3 even after 24 hours, likely due to differences in ketamine dosage and bupivacaine concentration. Local infiltration has also been utilized in spinal anesthesia patients [12].

According to a study by Singh and Prasad, postoperative morphine consumption and pain scores were significantly reduced in patients undergoing abdominal hysterectomy following local infiltration with dexmedetomidine (1 mcg/kg) and 0.25% bupivacaine. However, the study noted minimal sedation among participants [13]. Our findings with bupivacaine and dexmedetomidine infiltration were similar. In contrast, Group C showed a lower RSS at the sixth hour compared to Groups D and K, indicating that patients in Group C were less sedated and more agitated. This discrepancy may be attributed to the use of RSS in our study versus a four-point sedation scale in theirs.

Azemati et al. investigated the effects of adding dexmedetomidine to local bupivacaine infiltration for postoperative pain management in pediatric herniorrhaphy. They administered 0.2 ml/kg of 0.5% bupivacaine and 1 mcg/kg of dexmedetomidine at the incision site before surgery, while the control group received bupivacaine with normal saline. Their study, consistent with our findings, reported that the dexmedetomidine group experienced higher levels of drowsiness and analgesia compared to the control group [14]. We observed similar results with the combination of 0.25% bupivacaine and 1 mcg/kg of dexmedetomidine for local infiltration at the surgical site.

Surgeons may be hesitant to use local wound infiltration due to concerns about potential delayed wound healing. However, a study by Luan et al. found that patients who underwent open gastrectomy did not experience any adverse effects from adding 1.0 mcg/kg of dexmedetomidine to 0.3% ropivacaine for wound infiltration [15]. Similarly, our study observed that the addition of 1 mcg/kg of dexmedetomidine or ketamine to 0.25% bupivacaine for local infiltration at the wound site did not impact wound healing.

A primary limitation of our study was the use of a single dose of each drug during the postoperative phase, rather than preemptive infiltration. Additionally, the study was limited by the lack of literacy among some patients in this group.

## Conclusions

The analgesic effects of the local anesthetic bupivacaine are notably enhanced by the adjuvants dexmedetomidine and ketamine. Adding ketamine or dexmedetomidine to bupivacaine for local infiltration provides effective analgesia while also reducing patient agitation. Of the two adjuvants, ketamine offers superior patient satisfaction and analgesic relief compared to dexmedetomidine.
